# Gangrene in Diabetes

**Published:** 1867-03

**Authors:** 


GANGRENE IN DIABETES.—M. Verneuil, the eminent Paris surgeon, brought this subject a short time ago before the Surgical Society, and mentioned that he had met, in private and hospital practice, within the last six months, no less than half a dozen such cases. All the patients died.—*London Lancet.*

